# The Assembly of EDC4 and Dcp1a into Processing Bodies Is Critical for the Translational Regulation of IL-6

**DOI:** 10.1371/journal.pone.0123223

**Published:** 2015-05-13

**Authors:** Eri Seto, Reiko Yoshida-Sugitani, Toshihiko Kobayashi, Noriko Toyama-Sorimachi

**Affiliations:** 1 Department of Molecular Immunology and Inflammation, Research Institute, National Center for Global Health and Medicine, Tokyo, Japan; 2 Department of Virology and Preventive Medicine, Gunma University Graduate School of Medicine, Gunma, Japan; Toho University School of Medicine, JAPAN

## Abstract

Macrophages play critical roles in the onset of various diseases and in maintaining homeostasis. There are several functional subsets, of which M1 and M2 macrophages are of particular interest because they are differentially involved in inflammation and its resolution. Here, we investigated the differences in regulatory mechanisms between M1- and M2-polarized macrophages by examining mRNA metabolic machineries such as stress granules (SGs) and processing bodies (P-bodies). Human monocytic leukemia THP-1 cells cultured under M1-polarizing conditions (M1-THPs) had less ability to assemble oxidative-stress-induced SGs than those cultured under M2-polarizing conditions (M2-THPs). In contrast, P-body assembly in response to oxidative stress or TLR4 stimulation was increased in M1-THPs as compared to M2-THPs. These results suggest that mRNA metabolism is controlled differently in M1-THPs and M2-THPs. Interestingly, knocking down EDC4 or Dcp1a, which are components of P-bodies, severely reduced the production of IL-6, but not TNF-α in M1-THPs without decreasing the amount of IL-6 mRNA. This is the first report to demonstrate that the assembly of EDC4 and Dcp1a into P-bodies is critical in the posttranscriptional regulation of IL-6. Thus, improving our understanding of the mechanisms governing mRNA metabolism by examining macrophage subtypes may lead to new therapeutic targets.

## Introduction

Macrophages play fundamental roles not only in inflammation and host defense, but also in tissue remodeling and other homeostatic functions[1–3]. These cells exhibit phenotypic diversity and plasticity in response to various environmental factors, including cytokines and metabolites, and can change their activation phenotype to adapt to distinct environmental stimuli[2,4]. Classical (M1) activation by Interferon gamma (IFN-γ and lipopolysaccharide (LPS) generates macrophages with microbicidal effector functions and other pro-inflammatory properties[5]. In contrast, alternative (M2) activation in the presence of IL-4 and IL-13 generates macrophages with anti-inflammatory properties that are associated with tissue remodeling and the resolution of inflammation[6,7]. The dynamic changes in macrophage function strongly affect the onset of inflammatory conditions such as infection[8], allergy[9], tumor[10], diabetes[3], and arteriosclerosis[11]. Therefore, determining the precise nature of the unique regulatory mechanisms of polarized macrophages may lead to cell-type-specific therapeutic approaches that enhance host defense while preserving tissue integrity and preventing chronic inflammatory diseases.

Studies have revealed that the functional polarization of macrophages is intricately regulated through signaling events that are triggered by environmental stimuli and are followed by transcriptional events that induce a set of genes[4,12]. The epigenetic modulation of the chromatin states of various genes, such as those encoding transcription factors and cytokines, is also important for regulating macrophage polarization[13]. The signaling pathways and several of the functional molecules involved in these regulatory systems have been investigated extensively[14]. However, the properties of posttranscriptional regulation in polarized macrophages have received much less attention. In recent years, small non-coding RNAs, called microRNAs (miRNAs), have also emerged as important regulators of macrophage polarization and function[15].

Before miRNA can exert its functions, pre-miRNA must be cleaved by the endoribonuclease Dicer to produce mature miRNA, which is 20 to 25 bases in length[16]. Mature miRNA is assembled into a miRNA-induced silencing complex that helps to regulate mRNA stability. In miRNA-mediated posttranscriptional regulation, various RNA-binding proteins (RBPs) help to determine the fate of the mRNA. In eukaryotes, mRNAs form complexes with a wide variety of proteins in the cytoplasm, and an mRNA’s stability and translation are largely affected by the RBPs associated with it[17]. These mRNA and protein complexes (mRNPs), which also contain miRNA, form aggregates that can be microscopically identified as specific cytoplasmic foci, such as processing bodies (P-bodies)[18,19] and stress granules (SGs)[20,21]. SGs and P-bodies are highly dynamic, membraneless cytoplasmic granules observed in a variety of eukaryotic cells[17,22]. They affect mRNA stability, turnover, and subcellular localization, and are thus important in the translational regulation of gene expression[18–22]. SGs are observed when translation initiation is stalled during a stress response, and are composed largely of preassembled translation complexes that can be released rapidly to resume gene expression[23]. Therefore, SGs are thought to serve as temporary repositories for mRNAs. P-bodies contain enzymes involved in mRNA decay, such as decapping enzymes and exonucleases, and those required for mRNA degradation, particularly for active silencing via miRNA or RNAi mechanisms[18]. Although P-bodies are constitutively present in the steady state, they increase in size and number when translation is arrested[24]. SGs and P-bodies control mRNA metabolism through a rapid, highly dynamic process that is executed according to the specific biological context. Although the nature and regulatory mechanisms of SGs and P-bodies are largely unsolved, it is thought that these cytoplasmic structures are involved in regulating the final stage of gene expression, and that a dynamic cycle of mRNP compartmentalization and release among SGs, P-bodies, and polysomes strongly affects protein expression.

The stability and turnover of cytokine mRNAs during inflammation have been studied extensively[25,26]. A number of elegant studies have clarified the molecular mechanisms by which an individual RBP regulates a particular cytokine mRNA[27–30]. The tristetraprolin (TTP)-dependent translational regulation of TNF-α for example, is fairly well understood[31]. However, although TTP is a component in both SGs and P-bodies[32], no studies have been conducted to determine whether mRNA metabolism mediated by the SG and P-body machineries is regulated differently in M1 and M2 macrophages. Elucidating the differences between mRNA metabolic states in these cells may lead to novel strategies for manipulating macrophage subset-specific functions. We here show for the first time that the assembly of SGs and P-bodies under inflammatory conditions is regulated differently in macrophages depending on their state of activation and differentiation. Furthermore, we demonstrate that the P-body components mRNA-decapping enzyme 1a (Dcp1a) and enhancer of mRNA decapping 4 (EDC4) are crucial in the posttranscriptional regulation of IL-6. Our observations reveal a new aspect of cytokine regulation in functionally polarized macrophages.

## Materials and Methods

### Ethics statement

Protocols involving biohazards were reviewed and approved by the National Center for Global Health and Medicine biosafety committee.

### Cell culture

THP-1 human monocytic leukemia cells[33] were kind gifts from Dr. K. Miyake (University of Tokyo), and cultured in complete RPMI 1640 medium (Sigma-Aldrich, St. Louis, MO) supplemented with 10% FCS, 10 mmol/L HEPES, 2 mmol/L L-glutamine, 1 mmol/L sodium pyruvate, 50 μM 2-ME, 1% (v/v) non-essential amino acids, 100 U/mL penicillin, and 100 μg/mL streptomycin. The cells were differentiated by incubation for 16 hours in complete RPMI medium with PMA (192 ng/ml) (Sigma-Aldrich) for 18 hours. All cells were maintained at 37°C with 5% CO_2_.

### THP-1 polarization conditions

THP-1 cells were polarized as described previously[34,35]. Briefly, PMA-differentiated THP-1 cells were left untreated or were activated toward the M1 phenotype by culture in the presence of 20 ng/ml IFN-γ (PeproTech, EC Ltd., London, UK) plus 10 ng/ml LPS (from *E*. *coli* 055:B5, Sigma-Aldrich), or toward the M2 phenotype in the presence of 20 ng/ml IL-4 (PeproTech) plus 20 ng/ml IL-13 (PeproTech), for various time periods. Cells were placed in 24-well culture plates and exposed to one of the polarizing conditions or to buffer (control). Polarized phenotypes were identified by RT-PCR analysis of CXCL9, TNF-α, TGF-β, and IL-1ra mRNA.

### RNA interference

THP-1 cells were transfected with siRNAs (100 pmol/10^6^ cells) using the Neon transfection system (Invitrogen) according to the manufacturer’s instructions. SMARTpool siRNA against EDC4 (M-016635-00-0005), Dcp1a (M-021242-01-0005), and non-targeting siRNA pool #2 (D-001206-14-05) were purchased from Dharmacon (Chicago, IL). After transfection, the cells were seeded at an initial density of 10^6^ cells/ml and incubated for 24 hours before PMA stimulation.

### Immunohistochemical analyses

Differentiated THP-1 cells placed on coverslips in 24-well plates were washed twice with PBS, fixed with 4% paraformaldehyde for 15 minutes at room temperature, treated with PBS containing 0.1% Triton-X 100 for 5 minutes, and blocked with PBS containing 3% bovine serum albumin for 1 hour to prevent non-specific protein binding. The cells were then stained with primary antibodies diluted in blocking buffer for 1 hour at room temperature, using the following antibodies: anti-G3BP monoclonal (BD Biosciences, San Jose, CA, Cat# 611126), anti-TIA-1 polyclonal (Santa Cruz, CA, sc-1751), anti-EDC4 polyclonal (Santa Cruz, sc-137444), and anti-Dcp1a monoclonal (Abnova, Taipei, Taiwan, Cat# H00055802-M6). The cells were then washed three times with washing buffer (0.1% BSA, 0.05% azide in PBS) and stained with secondary antibodies conjugated to Alexa Fluor 488 and 568 (Invitrogen Life Technologies, Carlsbad, CA) for 1 hour. The cells were then washed, stained with Hoechst 33342 (Cell Signaling Beverly, MA), and mounted on glass slides with ProLong Gold Antifade Reagent (Molecular Probes). Images were acquired at RT with a confocal microscope (FV1000-D IX81 system, Olympus, Tokyo, Japan) equipped with a 60×/ 1.42 NA oil-immersion objective lens. The digitized images were processed using Adobe Photoshop software (San Jose, CA). To detect P-body formation, confocal images consisting of eight 1-μm slices along the z-axis were digitally merged. To quantify SGs and P-bodies, 30 cells were selected from multiple fields of view, and the number of fluorescence-positive dots was counted in each cell. To induce oxidative stress, cells were treated with 0.5 mM sodium arsenite (Sigma-Aldrich) for 30 minutes.

### Immunoblot analysis

Cells were harvested, washed with PBS, and lysed with RIPA buffer (50 mM Tris-HCl, pH 8.0, 150 mM NaCl, 1% NP-40, 0.5% sodium deoxycholate, and 0.1% SDS) supplemented with a cOmplete Protease Inhibitor Cocktail Tablet (Roche, Mannheim, Germany) and Halt Phosphatase Inhibitor Cocktail (Thermo Scientific Waltham, MA). Cell lysates were rotated for 40 minutes at 4° C, and centrifuged at 10,000 g for 10 minutes. The clarified cell extracts were assayed for protein concentration using DC Protein Assay Reagent (BioRad, Hercules, CA, Cat# 500-0116JA). The electrophoretically separated proteins were transferred to PVDF membranes, blocked with Blocking One (Nacalai Tesque, Inc., Kyoto, Japan), and probed with primary antibodies diluted with x20 Blocking One in 0.1% Tween 20 with PBS (PBS-T) at 4°C overnight. To detect TTP and Dcp2, anti-ZFP36 (TTP) polyclonal antibody (MBL, Nagoya, Japan, Cat# RN031PW) and anti-Dcp2 polyclonal antibody (Abcam, Cambridge, United Kingdom, Cat# ab28658) were used, respectively. The blots were washed with PBS-T, probed with HRP-conjugated secondary antibodies (Dako, Japan Inc., Tokyo, Japan) for 1 hour at room temperature, and washed again. Target proteins were detected with Supersignal West Femto Chemiluminescent Substrate (Thermo Scientific) and visualized using an ImageQuant LAS 4000mini (GE Healthcare Life Sciences, Buckinghamshire, UK). As an internal control, β-actin protein expression was assessed using a monoclonal anti-β-actin antibody purchased from Cell Signaling.

### RNA extraction, cDNA synthesis of mRNAs and miRNAs, and real-time quantitative PCR (qPCR)

Total RNA was isolated using Isogen (Nippongene, Tokyo, Japan) according to manufacturer’s instructions. For cDNA synthesis of mRNAs, 500 ng of total RNA was reverse-transcribed using Superscript III reverse transcriptase (Invitrogen) with random hexamer. 25 ng of cDNA aliquots from each sample were subjected to PCR reaction. The primers used in these standard PCR analyses are listed in S1 Table.

Quantitative real-time PCR analysis was performed using a StepOne Real-Time qPCR system (Applied Biosystems, Santa Clara, CA). Each 20 μl qPCR reaction contained 0.25 μM forward primer, 0.25 μM reverse primer, and 1 x Thunderbird SYBR qPCR mix (Toyobo Co., Ltd., Osaka, Japan). The primers used in qPCR analyses are listed in S2 Table. The qPCR reaction was performed in 48-well cluster plates at 95°C for 3 min, followed by 40 cycles of 95°C for 15 sec, 60°C for 30 sec, and 72°C for 30 sec with melting curve analysis (95°C for 1 min, 60°C for 30 sec, and 95°C for 30 sec). A standard curve was constructed using serial dilutions of cDNA from an arbitrary RNA set to 1.0, and a relative amount of each gene expression was calculated based on the standard curve. RNAs were normalized to HPRT RNA.

For qPCR of miRNAs, miRNA was converted to cDNA using Superscript III reverse transcriptase (Invitrogen) with the mixture of miRNA specific stem-loop primers (5′-GTTGGCTCTGGTGCAGGGTCCGAGGTATTCGCACCAGAGCCAACAACTAT-3′ for hsa-let7a, 5′-GTTGGCTCTGGTGCAGGGTCCGAGGTATTCGCACCAGAGCCAACCAGCTG-3′ for miR199, and 5′-GTTGGCTCTGGTGCAGGGTCCGAGGTATTCGCACCAGAGCCAACACCCCT -3′ for miR-155). Details for reverse transcription reaction were described previously[36]. Each miRNA was detected by the mature DNA sequence as the forward primer (S3 Table) and a 3′ universal reverse primer (5′- GTGCAGGGTCCGAGGT -3′). The qPCR reaction was conducted using Thunderbird SYBR qPCR mix (Toyobo Co., Ltd., Osaka, Japan) at 95°C for 10min, followed by 45 cycles of 95°C for 15sec, 60°C for 1min with 10 initial cycles of touchdown steps (70–60°C). The specificity of the reaction was verified by melting curve analysis. In some experiments, the qPCR reaction was conducted using TaqMan MicroRNA Reverse Transcription Kit (Applied Biosystems).

### Cytokine production

PMA-differentiated THP-1 cells that had been incubated for 8 hours under M1-polarizing conditions were stimulated with 1 μg/ml LPS from *E*. *coli* (Sigma-Aldrich). Supernatants were harvested at 0, 8, and 24 hours after stimulation, and were stored at -20°C until analysis. The IL-6 and TNF-α protein levels in the supernatants were measured with an ELISA kit (R&D Systems) according to the manufacturer’s instructions.

### Statistical analysis

Differences in values were analyzed for statistical significance by a two-tailed Student’s *t*-test. Differences with a *P-*value less than 0.05 were considered significant.

## Results

### SG assembly in THP-1 cells functionally polarized for different cytokine conditions

**Fig 1:**
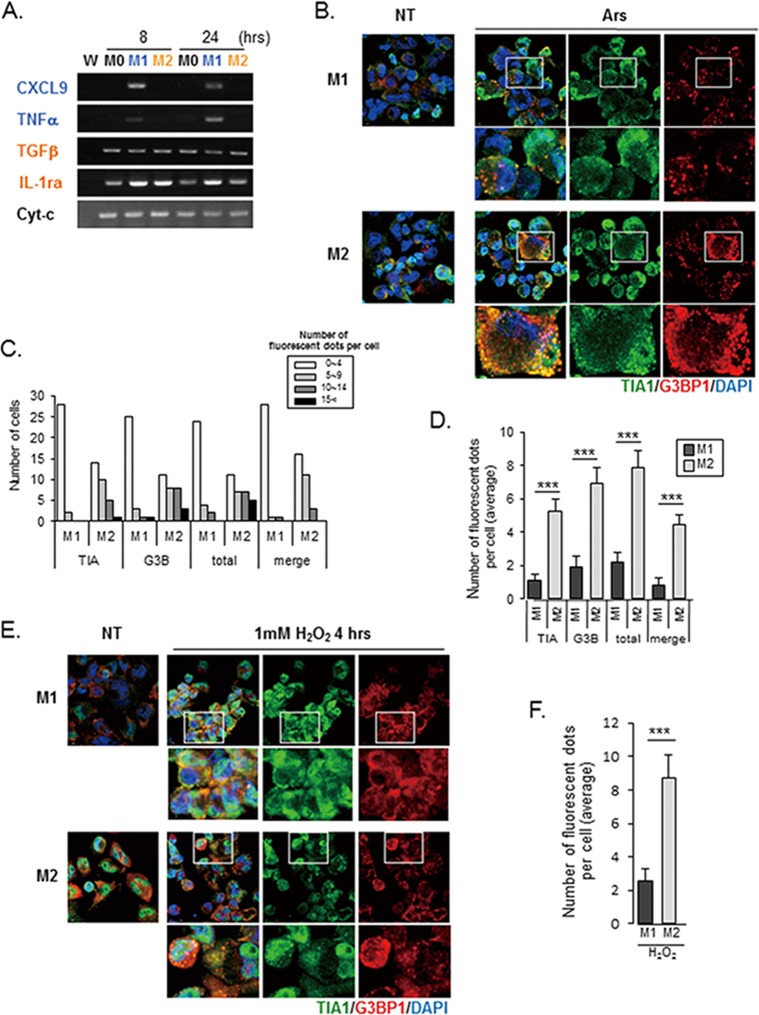
SG accumulation is induced by oxidative stress in M1- and M2-polarized macrophages. (A) The upregulation of M1 and M2 biomarkers in polarized PMA-differentiated THP-1 cells was confirmed by RT-PCR analysis. PMA-differentiated THP-1 cells were either left untreated (M0) or treated with LPS and IFN-γ (M1 activation) or IL-4 and IL-13 (M2 activation); RNA was isolated 8 and 24 hours after treatment. RT-PCR was performed to determine quantitative changes in mRNAs serving as activation biomarkers. W: no template control for PCR analysis. (B-F) SG formation in response to arsenite- or H_2_O_2_-induced oxidative stress was stronger in M2-THPs than in M1-THPs. (B) M1-THPs and M2-THPs, either untreated (NT) or treated with 0.5 mM arsenite (Ars) for 30 minutes, were fixed, stained with a mouse monoclonal antibody specific for G3BP1 and goat polyclonal antibody specific for TIA-1, and then stained with Alexa 488-conjugated donkey anti-goat IgG and Alexa 568-conjugated donkey anti-mouse secondary antibodies. The cells were stained with Hoechst 33342 to visualize nuclei, and the images were merged digitally. (C,D) Cells were left untreated or treated with arsenite, and the number of distinct cytoplasmic puncta positive for TIA1, G3BP1, TIA1, or G3BP1 (total), and both TIA1 and G3BP1 (merge) was counted for each cell type. ***p<0.001 (C) The number of fluorescent dots per cell was counted in 30 cells for each cell type, and the cells were categorized as shown in the bar graph. (D) The average number of dots positive for TIA1, G3BP1, TIA1 or G3BP1, and TIA1 and G3BP1 is shown for each cell type; dots were counted in 30 individual cells of each type. Error bars indicate SD. (E) SG formation in M1-THPs and M2-THPs, either untreated (NT) or treated with 1 mM H_2_O_2_ for 4 hours, was examined by immunofluorescence as in B. (F) Bar graph showing the average number of foci positive for both TIA1 and G3BP1 per cell. Error bars indicate SD. ***p<0.001

PMA-differentiated THP1 cells were incubated under M1 (IFN-γ plus LPS) or M2 (IL-4 plus IL-13) conditions, and the resulting M1- or M2-like phenotypes were verified by detecting the transcription of selected cytokines and chemokines as biomarkers. M1-THPs transcribed the CXCL9 and TNF-α genes after LPS stimulation (Fig 1A). In M2-THPs, the CXCL9 and TNF-α gene expressions were not detected, while the TGF-β and IL-1ra genes were transcribed, and the level of TNF-α secreted after LPS stimulation was low compared to that in M1-THPs (Figs 1A and 6D).

Using these polarized THP-1 cells, we examined whether the SG formation in macrophages was influenced by their functional polarization. We used sodium arsenite, which strongly induces oxidative stress, to efficiently induce SG formation[37]. When M1-THPs and M2-THPs were treated with arsenite, we observed that T-cell intracellular antigen 1 (TIA1) and Ras-GAP SH3 domain binding protein 1 (G3BP1), which are essential components for assembling SGs[20], localized to cytoplasmic foci (Fig 1B). A count of cytoplasmic dots stained with TIA1 and/or G3BP1 showed that M1-THPs had less ability than M2-THPs to form SGs (Fig 1C and 1D). The results were similar when the cells were treated with H_2_O_2_; staining for TIA1 and G3BP1 showed dot-like structures in M2-THPs, but there was no visible accumulation of TIA1 and G3BP1 at cytoplasmic foci in M1-THPs (Fig 1E and 1F). Since the appearance of SGs is inversely related to the persistence of translation[38], these results suggest that M1-THPs have a lower incidence of translational repression in the presence of oxidative stress than M2-THPs.

### Differential regulation of P-body assembly in M1- and M2-conditioned THP1 cells

**Fig 2:**
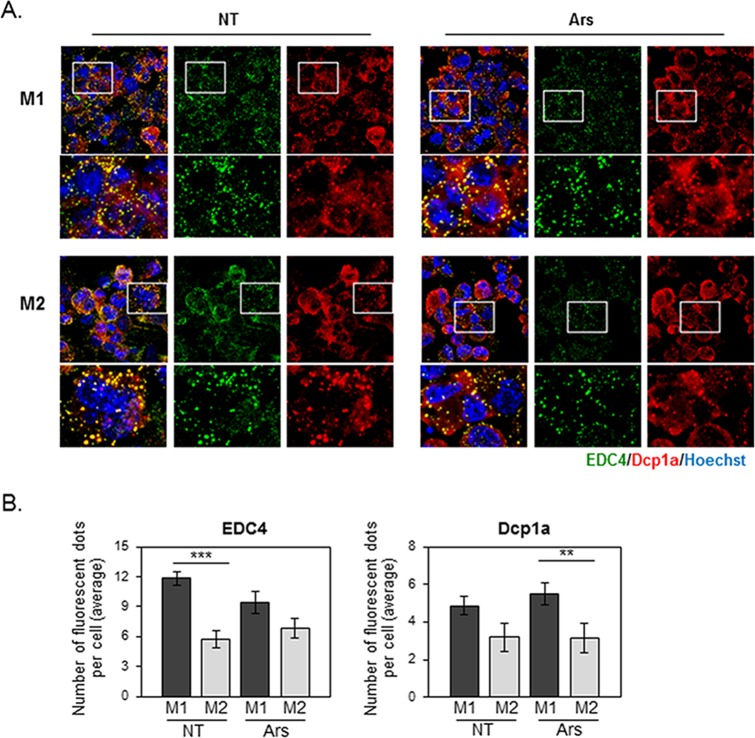
Oxidative stress does not induce P-body accumulation in M1-THPs or M2-THPs. (A) M1- and M2-polarized THP-1 cells, either untreated (NT) or treated with 0.5 mM arsenite (Ars) for 30 minutes, were fixed and stained with a goat polyclonal antibody specific for EDC4 and a mouse monoclonal antibody specific for Dcp1a, followed by staining with Alexa 488-conjugated donkey anti-goat IgG and Alexa 568-conjugated donkey anti-mouse secondary antibodies. (B) Bar graph showing the average number of EDC4- or Dcp1a- positive foci per cell. The fluorescent dots were counted as described in Methods, and the error bars indicate SD. **p<0.01, ***p<0.001

We next compared oxidative stress-induced P-body formation in M1-THPs and M2-THPs by staining with antibodies against Dcp1a or EDC4, key marker proteins that are linked to P-body formation[39,40]. EDC4-positive cytoplasmic foci were present in both M1-THPs and M2-THPs, and most of these were also positive for Dcp1a (Fig 2A). Although arsenite treatment did not increase the number of P-bodies in M1-THPs or M2-THPs, the M1-THPs were able to assemble more P-bodies than M2-THPs whether in the presence or absence of arsenite stimulation (Fig 2B). These results suggested that the regulation of P-body assembly differed in M1-THPs and M2-THPs.

**Fig 3:**
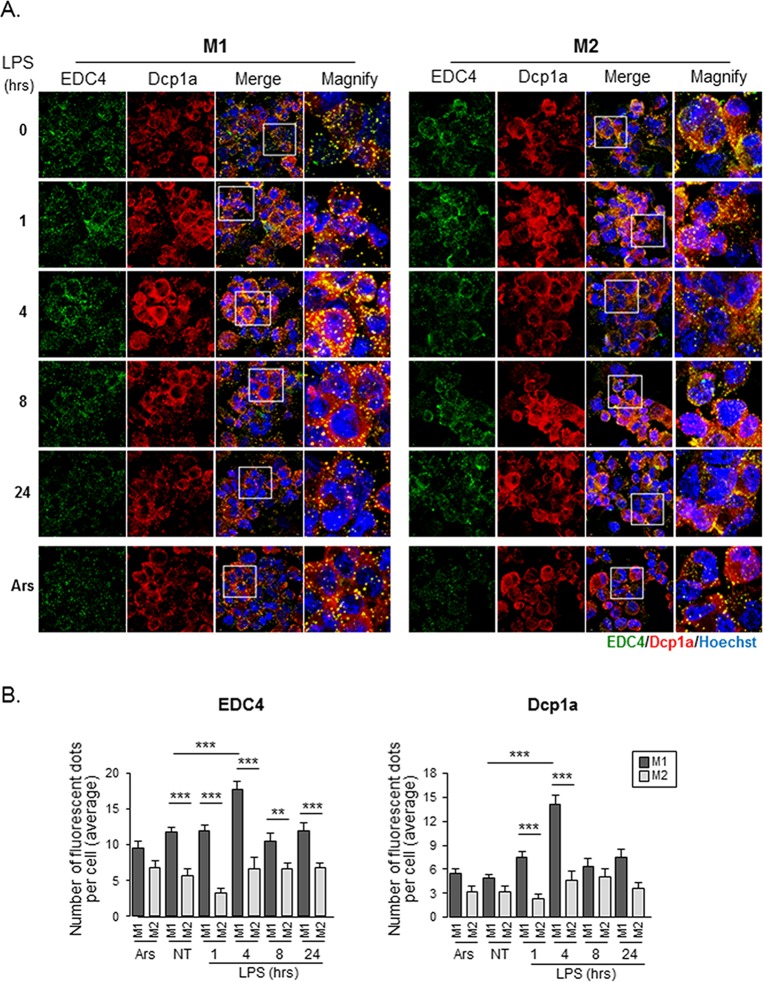
LPS-stimulated M1-THPs accumulate P-bodies. (A) M1-THPs and M2-THPs were either untreated or treated with 1 μg/ml LPS for 1, 4, 8, or 24 hours. Cells were fixed and processed for immunofluorescence to detect P-body accumulation as described in Fig 2. (B) Bar graph showing the average number of EDC4- or Dcp1a-positive foci per cell. Error bars indicate SD. **p<0.01, ***p<0.001

We then asked whether the machinery regulating P-body assembly differed between M1- and M2-polarized macrophages under inflammatory conditions. Antibodies against EDC4 and Dcp1a stained discrete cytoplasmic foci in LPS-stimulated M1-THPs, and the areas stained by these antibodies largely overlapped (Fig 3A). The number and size of the foci appeared to be transiently increased in the M1-THPs 4 hours after LPS stimulation. In contrast, M2-THPs showed diffuse cytoplasmic staining for Dcp1a, and although EDC4-positive foci were observed, the edges were poorly defined, and EDC4 was partly distributed in the cytoplasm. Notably, a count of Dcp1a- and EDC4-positive foci showed that the P-body assembly was more robust in M1-THPs than in M2-THPs, and that LPS stimulation enhanced the P-body formation in M1-THPs (Fig 3B). In M2-THPs, the number of P-bodies within a cell did not change substantially after LPS stimulation.

### Expression of SG and P-body components in polarized THP1 cells

**Fig 4:**
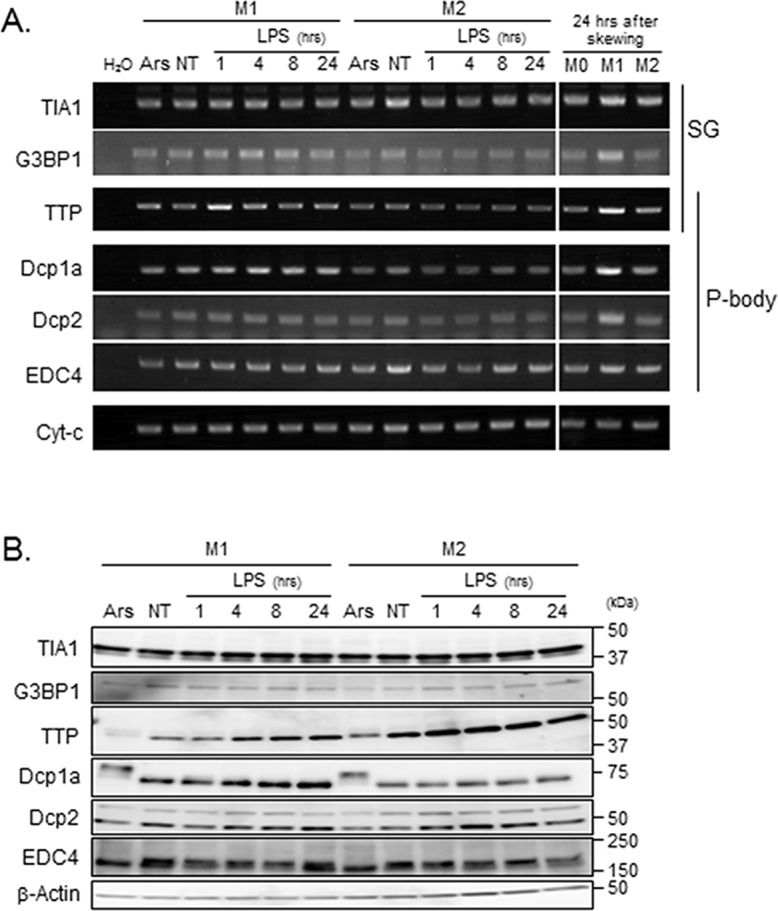
SG and P-body components are expressed in M1-THPs and M2-THPs. (A) M1-THPs and M2-THPs with no treatment (NT) or stimulated with LPS for 1, 4, 8, or 24 hours were harvested and subjected to semi-quantitative RT-PCR (A) and immunoblot analyses using antibodies against the SG or P-body components indicated; (B) β-actin served as a loading control.

We next compared the expression levels of various SG and P-body components in M1-THPs and M2-THPs. We found no obvious differences in TIA1 or G3BP1 expression at either the transcriptional or protein level (Fig 4A and 4B), and the expression levels of these SG components were not affected by oxidative stress or by LPS stimulation. The levels of TTP, a zinc-finger-containing protein that promotes SG nucleation and the decay of AU-rich region (ARE)-containing mRNAs at P-bodies[32], increased in both M1-THPs and M2-THPs upon LPS stimulation. The most noticeable difference between M1-THPs and M2-THPs was in the expression levels of Dcp1a, an essential P-body component that also forms part of the decapping complex[22]. The Dcpla protein and mRNA levels were higher in M1-THPs than M2-THPs, and their expression in M1-THPs gradually increased during LPS stimulation (Fig 4A and 4B). This increase of Dcp1a in M1-THPs was consistent with the increased number of P-bodies in M1-THPs. When cells were treated with arsenite, a slow-migrating Dcp1a band was observed in both M1-THPs and M2-THPs (Fig 4B). This probably corresponded to a phosphorylated Dcp1a, since Dcp1a is known to undergo hyper-phosphorylation[41]. A slow-migrating band was not observed in LPS-treated cells. The protein level of Dcp2, a P-body component that catalyzes cytoplasmic mRNA decapping in conjunction with its coactivator Dcp1a[42], was similar in M1-THPs and M2-THPs.

### Dcp1a- and EDC4-dependent posttranslational regulation of IL-6

**Fig 5:**
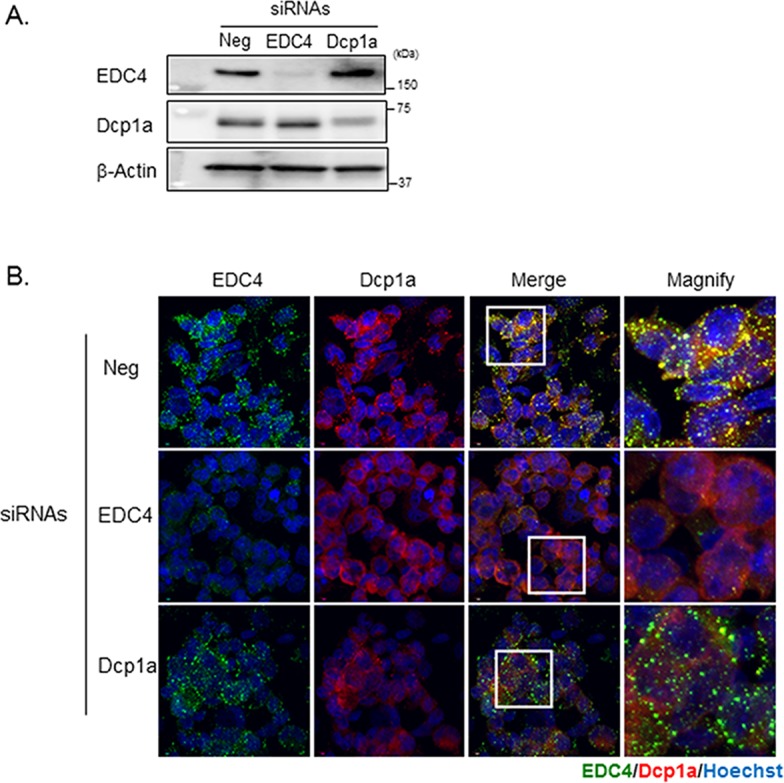
EDC4 or Dcp1a knockdown affects P-body assembly. THP-1 cells were transfected with the indicated siRNAs; 20 hours after transfection, the cells were PMA-differentiated and then activated toward a M1 phenotype. (A) The depletion of EDC4 or Dcp1a by siRNA. The siRNA-transfected M1-THP cells were harvested and analyzed by immunoblotting with the indicated antibodies; β-actin was used as a loading control. (B) EDC4 depletion inhibited P-body formation. The siRNA-transfected cells were polarized toward an M1 phenotype, fixed, stained with goat polyclonal antibody specific for EDC4 and mouse monoclonal antibody specific for Dcp1a, and finally stained with Alexa 488-conjugated donkey anti-goat and Alexa 568-conjugated donkey anti-mouse secondary antibodies.

To determine whether the preferential localization of Dcp1a and EDC4 to P-bodies in M1 macrophages has a functional role during the cell's response to LPS stimulation, we examined the effect of EDC4 or Dcp1a knockdown on LPS-triggered cytokine production. Knockdown efficiency was confirmed by western blotting, as shown in Fig 5A. EDC4 knockdown caused most of the EDC4-positive and Dcp1a-positive cytoplasmic foci to disappear, indicating that the EDC4 loss disrupted P-body assembly (Fig 5B). Interestingly, Dcp1a knockdown resulted in an obvious loss of Dcp1a-positive but not EDC4-positive foci; this suggested that EDC4 facilitates the assembly of a different type of P-body in the absence of Dcp1a, although such EDC4-positive foci might differ functionally from P-bodies containing both EDC4 and Dcp1a.

**Fig 6:**
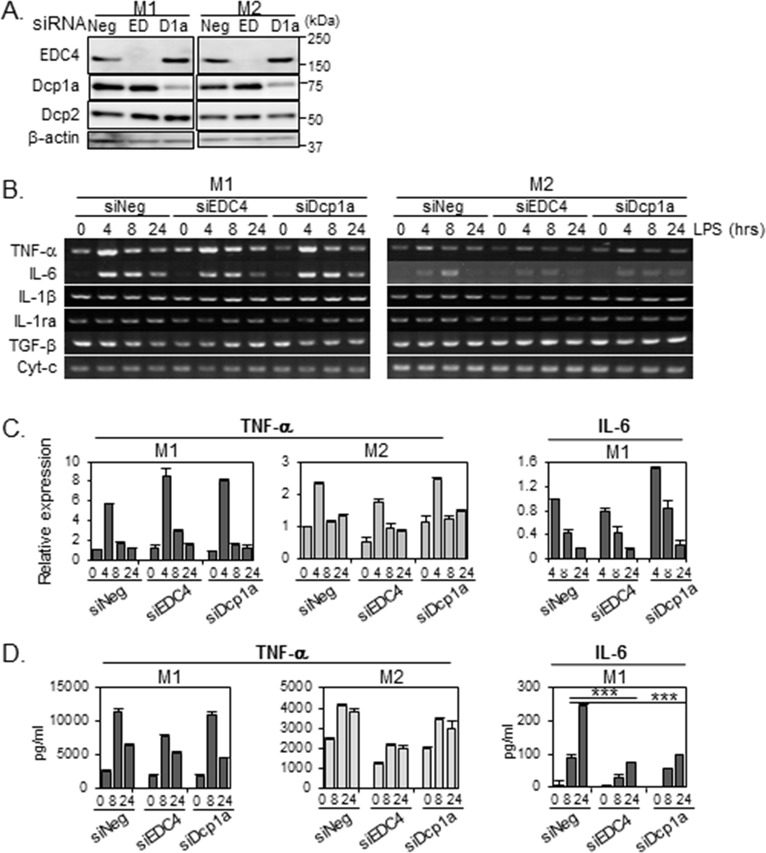
P-body knockdown reduces LPS-stimulated IL-6 release in M1-THPs. THP-1 cells were transfected with siRNA targeting EDC4 (EDC), Dcp1a (D1a), or with a non-targeting siRNA (siNeg). Twenty hours after transfection, the cells were PMA-differentiated, activated toward an M1 or M2 phenotype, and subjected to LPS stimulation as described in Methods. (A) Depletion of EDC4 or Dcp1a by siRNA. The siRNA-transfected cells were polarized toward an M1 or M2 phenotype, harvested, and subjected to immunoblot analysis with the indicated antibodies; β-actin served as a loading control. (B) Effects of EDC4 or Dcp1a knockdown on cytokine mRNA expression. The siRNA-transfected cells were polarized toward an M1 or M2 phenotype, treated with LPS, harvested at the time points indicated, and analyzed by semi-quantitative RT-PCR. (C) P-body knockdown did not affect the levels of LPS-induced IL-6 and TNF-α mRNA expression in M1-THPs. Total RNA was isolated 0, 4, 8, or 24 hours after LPS stimulation and subjected to quantitative real-time RT-PCR analysis to detect IL-6 and TNF-α gene transcripts, as described in Methods. RNAs were normalized to HPRT RNA. The level of the IL-6 and TNF-α transcripts in siNeg-transfected cells at 4 and at 0 hours after LPS stimulation, respectively, was set to 1 to calculate the relative values in each sample. The IL-6 mRNA level before LPS stimulation (0 hour) was below the limit of detection. Data are averages of three independent experiments; error bars indicate SD. (D) P-body knockdown reduced the level of IL-6 released from M1-THPs cells. Supernatants were harvested at 0, 8, or 24 hours after LPS stimulation, and protein concentrations of IL-6 and TNF-α were measured by ELISA. IL-6 protein levels before LPS stimulation (0 hour) were below the limit of detection. Data are averages of three independent experiments; error bars indicate SD. ***p<0.001

We next examined the effect of EDC4 or Dcp1a loss on inflammatory responses in polarized THP-1 cells. We knocked down EDC4 or Dcp1a in M1-THPs and M2-THPs (Fig 6A), and stimulated the cells with LPS. The expression levels of cytokine genes in the LPS-stimulated cells were confirmed by RT-PCR (Fig 6B). LPS induced TNF-α and IL-6 transcription in both M1-THPs and M2-THPs, although the transcription was relatively weak in M2-THPs. Quantitative analyses showed that neither the TNF-α nor IL-6 mRNA levels were severely affected by EDC4 or Dcp1a knockdown (Fig 6C). Interestingly, however, the ELISA analysis of cytokine secretion showed that EDC4 or Dcp1a knockdown severely impaired the IL-6 secretion by M1-THPs (Fig 6D). IL-6 secretion by M2-THPs was not detectable by ELISA analysis (data not shown). Similar results were obtained in U937 leukemia cells under M1-skewing conditions, in which a decrease in EDC4 or Dcp1a expression suppressed the IL-6 production without severely altering the mRNA level (S1 Fig). In M2-skewed U937 cells, although the amount of secreted IL-6 protein was substantially less than in by M1-U937 cells, it was still measurable, and was also decreased by EDC4 or Dcp1a knockdown (S1 Fig). In addition, the decreased IL-6 production by EDC4 or Dcp1a knockdown in M2-U937 was accompanied by a decrease in IL-6 mRNA, suggesting that IL-6 production is regulated differently in M1- and in M2-U937 (S1 Fig). These results strongly suggest that the assembly of EDC4 and Dcp1a into P-bodies is critical for IL-6 production at the posttranscriptional level. The TNF-α secretion was partially decreased in M2-THPs, and to a lesser extent in M1-THPs, by EDC4 knockdown. TNF-α production was not decreased by Dcp1a knockdown, indicating that an EDC4-dependent but Dcp1a-independent machinery is involved in the posttranscriptional regulation of TNF-α.

### Dcp1a- and EDC4-dependent control of various IL-6 regulatory elements

**Fig 7:**
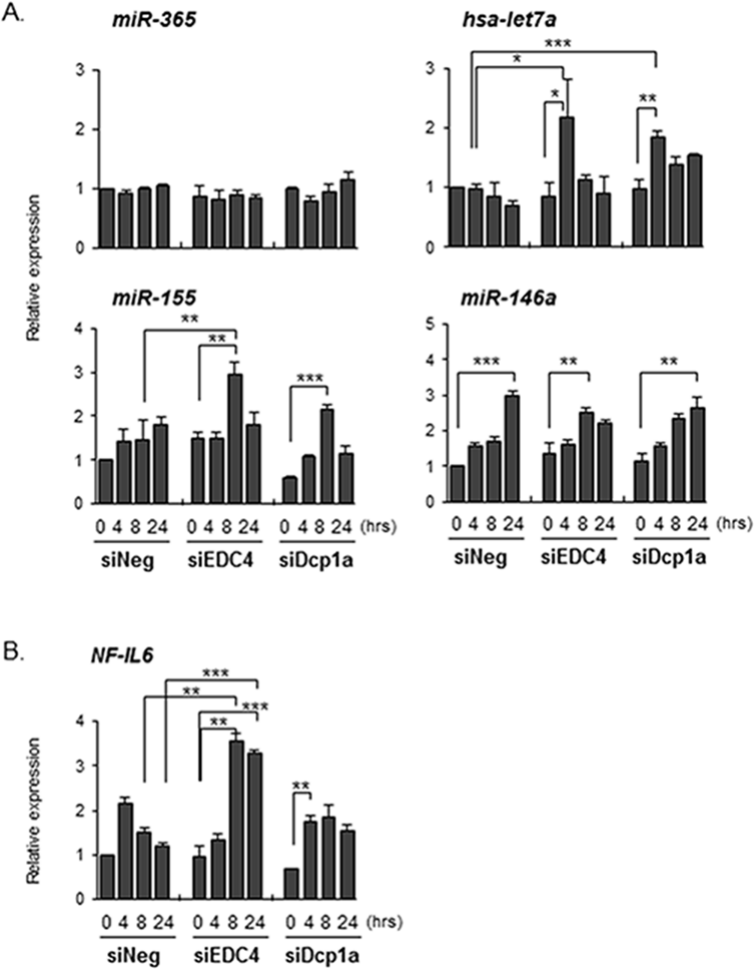
P-body knockdown affects the expression of IL-6-regulating molecules. THP-1 cells were transfected with a siRNA targeting EDC4 or Dcp1a, or with a non-targeting siRNA (siNeg). Twenty hours after transfection, the cells were differentiated with PMA, followed by activation later. The expression levels of (A) miRNAs and (B) NF-IL6 were determined by quantitative PCR as described in Methods. *p<0.05, **p<0.01, ***p<0.001

Our results of the EDC4 and Dcp1a knockdown experiments raised the possibility that negative regulators of IL-6 translation were controlled in a P-body-dependent manner. Therefore, we next examined the expression of molecules that are known to negatively regulate IL-6 production. It has been reported that miR-365 decreases IL-6 expression by repressing mRNA translation, without affecting IL-6 mRNA levels[43]. The expression of miR-365, however, was not changed by EDC4 or Dcp1a knockdown (Fig 7A). We next evaluated the expression levels of let-7a micro RNA, which is reported to inhibit IL-6 expression by binding the IL-6 3’-UTR directly[44,45]. Human let-7a microRNA has also been shown to repress protein translation by inhibiting actively translating polyribosomes[46]. The transcription of let-7a microRNA in control siRNA-treated M1-THPs tended to decrease 24 hours after LPS stimulation. However, LPS stimulation increased rather than decreased the let-7a microRNA in Dcp1a-knockdown M1-THPs (Fig 7A), and EDC4-knockdown M1-THPs also tended to show an increase in let-7a microRNA after LPS stimulation. These results suggested that the synthesis of let-7a microRNA is also controlled by P-body-dependent machinery, and that the inhibition of IL-6 production in EDC4- or Dcp1a-knockdown cells is partly due to an increase in let-7a microRNA.

We also examined whether Dcp1a and EDC4 contributed to transcripts such as NF-IL-6 and miR-155; both of these are known to regulate IL-6, and their expression is controlled through a decapping-dependent mechanism[47,48]. Interestingly, EDC4 knockdown augmented the miR-155 expression (Fig 7A and 7B). NF-IL6 transcription was also increased in EDC4-knockdown cells compared with cells treated with control siRNA or siDCP1a. We also examined miR-146a, which negatively regulates the NF-κB pathway by targeting IRAK1 and TRAF6[48,49]. However, disrupting Dcp1a or EDC4 did not affect miR-146a expression. These results reveal that IL-6 production is controlled by multiple elements at both the transcriptional and translational levels, and that the P-body components Dcp1a and EDC4 contribute differentially to this intricate regulatory machinery.

## Discussion

We investigated differences in the RNA metabolic machineries such as SGs and P-bodies in M1- or M2-polarized human macrophage THP-1 cells, and demonstrated that the control of SGs and P-bodies differs between M1-THPs and M2-THPs. In the presence of oxidative stress, SG formation was observed in M2-THPs but was hardly detectable in M1-THPs. On the other hand, P-body formation was augmented in M1-THPs both in the steady-state and upon TLR4 stimulation. These results suggest that RNA metabolism is controlled differently in different macrophage subsets, even in the presence of the same stimuli. Although a number of studies have investigated the functions of various microRNAs and shown their importance in regulating inflammatory responses[48,50], extensive comparative analyses of the RNA metabolic machinery in functionally different macrophage subtypes have not been performed. This is the first report to demonstrate that differentially polarized macrophage subsets differ in their ability to form SGs and P-bodies.

Dcp1a, a commonly used P-body marker, associates with EDC4 in P-bodies to co-activate the Dcp2 mRNA decapping enzyme[22,42]. Our siRNA-mediated knockdown experiments demonstrated that EDC4 is critical for P-body assembly; a decrease in EDC4 protein efficiently interrupted the assembly of Dcp1a into cytoplasmic foci. In contrast, EDC4 assembled and formed P-body-like foci (EDC4-bodies) even in the absence of Dcp1a. Therefore, it is possible that EDC4 functions as a scaffolding protein independently of Dcp1a, and that EDC4-bodies are functionally different from P-bodies that contain both EDC4 and Dcp1a. Recent studies demonstrated that P-bodies exhibit dynamic interactions with SGs such as docking and fusion[22,51]. Therefore, it should be noted that P-bodies and SGs are dynamic and heterogeneous structure. Although heterogeneity of P-bodies and SG in the macrophage subsets remain an important unresolved issue, our observation that knocking down either Dcp1a or EDC4 suppressed IL-6 production without an apparent reduction in IL-6 mRNA demonstrates that both Dcp1a and EDC4 are necessary for the production of IL-6 protein. This finding also suggests that the assembly of decapping machinery into P-bodies is important for IL-6 production.

Because the formation of the mRNA decapping complex is inversely related to the persistence of translation[38], it has been thought that disrupting the decapping machinery might inhibit mRNA degradation and thus increase protein synthesis. However, the loss of Dcp1a or EDC4 severely inhibited the production of IL-6 protein without noticeably decreasing its mRNA. Our results suggest at least two non-mutually exclusive possibilities: One is that Dcp1a and EDC4 are involved in the mRNA decay of negative regulators of IL-6 mRNA translation, and this possibility is supported by the increase of let7a miRNA in EDC4- or Dcp1a-knockdown cells. The other is that Dcp1a is required for the translational regulation of IL-6 mRNA. Previous studies have suggested that the sorting of mRNPs within P-bodies determines whether an mRNA is to be stored, degraded, or returned to translation[18,22,52]. Therefore, the assembly of P-bodies containing both Dcp1a and EDC4 may be necessary to cause IL-6 mRNA to be transferred to the translation initiation complex. Although the precise mechanism should be investigated further, our data revealed that the formation of P-bodies containing Dcp1a and EDC4 is a critical step in IL-6 production.

Our qPCR analyses indicated that, at least under our experimental conditions, disrupting decapping complexes by Dcp1a or EDC4 knockdown did not severely decrease the TNF-α or IL-6 mRNA levels in LPS-stimulated THP-1 cells. Although we did not evaluate the synthesis and degradation rates of these cytokine mRNAs, our observations strongly suggest that the decapping complex itself was not essential for maintaining the mRNA levels of these cytokines at the time points examined.

Macrophages are functionally and morphologically diverse, and change their functional phenotype in response to environmental stimuli. Our results raised the possibility that SG- or P-body-dependent posttranscriptional regulation of inflammation-related gene expression could be involved in phenotypic divergences of macrophages. In this context, the dramatic difference in M1-THPs and M2-THPs in the formation of SGs in the presence of oxidative stress is interesting. Since SGs are thought to regulate RNA degradation and translation[20,21], the repression of SG formation in M1-THPs may suggest that M1 macrophages are more resistant than M2 macrophages to translational repression under oxidizing conditions. Although the molecular mechanisms underlying the assembly of SGs and P-bodies are still largely unknown, an investigation of context-dependent posttranscriptional regulation in diverse macrophage subsets may lead to methods for fine-tuning inflammatory responses.

It should be noted that the metabolism of let7a and miR155 miRNA was controlled by EDC4 and, to a lesser extent, by Dcp1a. Our data also showed that the quantitative regulation of NF-IL6 mRNA was dependent on EDC4, particularly in the late phase of LPS stimulation. Thus, the production of IL-6 appears to be regulated by processes that are far more complex than previously thought.

## Supporting Information

S1 Fig P-body knockdown reduces the LPS-stimulated IL-6 release in U937 leukemia cells. U937 cells were transfected with siRNA targeting EDC4, Dcp1a, or with a non-targeting siRNA (siNeg). Twenty hours after transfection, the cells were PMA-differentiated, polarized toward an M1 or M2 phenotype, and subjected to LPS stimulation as described in Methods. (A) Depletion of EDC4 or Dcp1a by siRNA. Total RNA from EDC4- or Dcp1a-silenced cells polarized toward an M1 or M2 phenotype and followed by LPS stimulation for 24 hours was subjected to quantitative RT-PCR analysis. Expression levels were normalized to HPRT RNA. (B) P-body knockdown did not affect the level of LPS-induced IL-6 mRNA expression in M1-polarized U937 cells. Total RNA from M1- or M2-polarized cells stimulated with LPS for 24 hours was isolated and subjected to quantitative RT-PCR analysis to detect IL-6 transcripts, as described in Methods. Expression levels were normalized to HPRT RNA. The level of IL-6 transcript in siNeg-transfected cells at 0 hours after LPS stimulation was set to 1, and the relative value in each sample was calculated. Data are representative of two independent experiments; error bars indicate SD. (C) P-body knockdown reduced the amount of IL-6 protein released from both M1- and M2- polarized U937 cells. The IL-6 protein level in the culture supernatant of the M1- or M2-polarized U937 cells stimulated with LPS for 0 or 24 hours was measured by ELISA. Data are representative of two independent experiments; error bars indicate SD. N.D.; not detected.
(TIF)

S1 Table PCR primers used in the standard RT-PCR analyses. (XLSX)

S2 Table PCR primers used in the qPCR analyses. (XLSX)

S3 Table PCR primers used in the qPCR analyses of miRNA. (XLSX)
