# Supplementary figures and images for: The Assembly of EDC4 and Dcp1a into Processing Bodies Is Critical for the Translational Regulation of IL-6
Source: PLoS One. 2015 May 13;10(5):e0123223. doi: 10.1371/journal.pone.0123223 (PMC4430274; doi:10.1371/journal.pone.0123223)

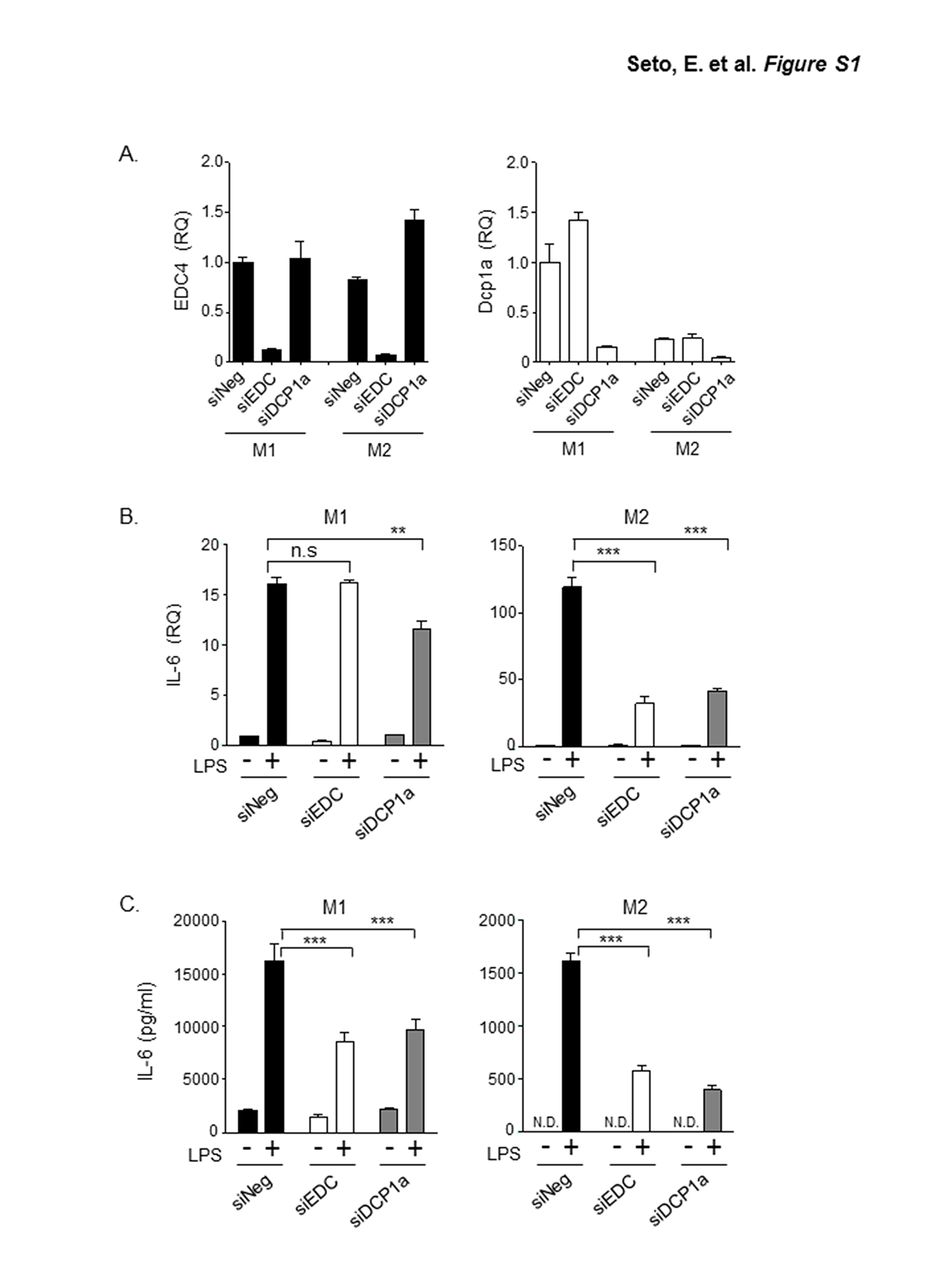

Supplement: S1 Fig — U937 cells were transfected with siRNA targeting EDC4, Dcp1a, or with a non-targeting siRNA (siNeg). Twenty hours after transfection, the cells were PMA-differentiated, polarized toward an M1 or M2 phenotype, and subjected to LPS stimulation as described in Methods. (A) Depletion of EDC4 or Dcp1a by siRNA. Total RNA from EDC4- or Dcp1a-silenced cells polarized toward an M1 or M2 phenotype and followed by LPS stimulation for 24 hours was subjected to quantitative RT-PCR analysis. Expression levels were normalized to HPRT RNA. (B) P-body knockdown did not affect the level of LPS-induced IL-6 mRNA expression in M1-polarized U937 cells. Total RNA from M1- or M2-polarized cells stimulated with LPS for 24 hours was isolated and subjected to quantitative RT-PCR analysis to detect IL-6 transcripts, as described in Methods. Expression levels were normalized to HPRT RNA. The level of IL-6 transcript in siNeg-transfected cells at 0 hours after LPS stimulation was set to 1, and the relative value in each sample was calculated. Data are representative of two independent experiments; error bars indicate SD. (C) P-body knockdown reduced the amount of IL-6 protein released from both M1- and M2- polarized U937 cells. The IL-6 protein level in the culture supernatant of the M1- or M2-polarized U937 cells stimulated with LPS for 0 or 24 hours was measured by ELISA. Data are representative of two independent experiments; error bars indicate SD. N.D.; not detected. (TIF) [file pone.0123223.s001.tif]
